# SG-APSIC1154: Evaluation of disposable antimicrobial curtains in an ambulatory cancer center

**DOI:** 10.1017/ash.2023.60

**Published:** 2023-03-16

**Authors:** Sin Hui Wong, Swee Peng Yap, Ming Zhen Priscilla Han

**Affiliations:** National Cancer Centre Singapore, Singapore; National Cancer Centre Singapore, Singapore; National Cancer Centre Singapore, Singapore

## Abstract

**Objectives:** The prevention of nosocomial infection is a challenge for all healthcare institutions. Privacy curtains are often changed infrequently, and they are difficult to clean. Contaminated curtains can be touched by healthcare providers and patients, which may result in indirect transmission of infectious disease. Hence, we evaluated the impact of the antimicrobial properties of disposable curtains and their cost-effectiveness. **Methods:** This descriptive exploratory study was conducted in an ambulatory cancer center in 2017. Privacy curtains were assigned to 2 cohorts, labelled E1 and E2. They were placed in the clinical areas for 6–12 months. Moist swab samples for MRSA, VRE, and CP-CRE cultures were obtained from the leading edges of the curtains during the evaluation period. Also, 10-cm × 10-cm swatches were cut from the high-touch areas of curtains and were tested for total aerobic count on the first of the month and quarterly thereafter. **Results:** All bacterial culture swabs obtained from the E1 and E2 cohorts of curtains were negative. The total bacterial plate count results from E1 curtains were negative for up to 1 year. However, the total bacterial plate count results for E2 curtains were positive in the sixth month. Using disposable curtains yielded an annual cost saving of ~50%. **Conclusions:** The use of appropriate impregnated antimicrobial disposable curtains can improve patient safety in the clinical areas. These curtains may eliminate potential sources of infection and thereby decrease the rate of nosocomial infection. They also save significant institutional costs by reducing frequent laundry and manpower requirements needed for the installation of curtains.

